# Establishment of qualitative human immunodeficiency virus type 1 nucleic acid amplification test as an adjunct confirmatory test in low-prevalence areas and small- and medium-sized diagnostic laboratories

**DOI:** 10.1016/j.heliyon.2024.e24451

**Published:** 2024-01-13

**Authors:** Shigeru Kusagawa, Ai Kawana-Tachikawa, Saori Matsuoka

**Affiliations:** AIDS Research Center, National Institute of Infectious Diseases, Japan

**Keywords:** HIV-1 antigen-positive case, HIV confirmatory test, Qualitative HIV-1 NAT, Fluorescent signal, Agarose gel electrophoresis, Limit of detection

## Abstract

Two simple and inexpensive in-house qualitative human immunodeficiency virus type 1 nucleotide amplification tests (HIV-1 NATs) were established as adjunct confirmatory HIV test for HIV antigen (Ag)-positive specimens identified from HIV screening test and for patients with indeterminate or negative HIV antibody (Ab) confirmatory test results. The limit of detection was <1000 copies/mL, which is lower than that of the HIV Ag/Ab combination assay. One test using QL1 detected all 11 HIV-1 subtypes/circulating recombinant forms/group samples with almost equal analytical sensitivity, and the other test, using QL2, also detected all, except for two group O samples. In the examination of 28 HIV-1 Ag-positive samples using Determine HIV Early Detect, 27 samples were reactive and one HIV-1 Ag-pseudo-positive sample was non-reactive using both methods. These in-house qualitative HIV-1 NATs are useful for confirming HIV-1 Ag-positive cases and excluding HIV-1 Ag false-positive cases in areas with low HIV prevalence and small- and medium-sized diagnostic laboratories.

## Highlights

• Immunochromatography assay is important for expansion of HIV test opportunities.
• Immunochromatography assay detects anti-HIV antibody and HIV-1 antigen separately.
• Inexpensive HIV confirmatory test for HIV-1 antigen-positive cases is required.
• Limit of detection of conventional RT-PCR is enough for HIV-1 antigen-positive cases.
• Conventional RT-PCR is accustomed for small- and medium-sized diagnostic laboratories.

## 1 Introduction

In 2021, 742 human immunodeficiency virus (HIV)-infected patients (without acquired immunodeficiency syndrome [AIDS]) and 315 AIDS patients were newly reported by the AIDS Surveillance Committee in Japan [1]. A total of 903 and 333 patients were reported in 2019 and 750 and 345 patients in 2020 with HIV and AIDS, respectively [1]. A decrease in the number of HIV-infected patients has been observed since 2020. Early diagnosis and treatment are important to control the HIV-1 epidemic. Free, anonymous HIV tests are prevalent in public health centers and prefectural diagnostic laboratories in Japan, which identify approximately 40 % of all HIV-infected patients every year [1]. A total of 54,551 HIV consultations (129,695 in 2019 and 66,519 in 2020) and 58,172 diagnostic tests (142,260 in 2019 and 68,998 in 2020) were performed in 2021; marked decreases in both were observed during the coronavirus disease 2019 (COVID-19) pandemic [1]. These results indicate the importance of their role in determining the extent of HIV infection status in Japan.

Rapid HIV diagnostic test using an immunochromatography assay (ICA) is an important tool for anonymous HIV test, and HIV testing in low-HIV prevalence areas and small- and medium-sized diagnostic laboratories with respect to cost. Determine HIV Early Detect (Abbott Diagnostic Medical, Matsudo, Japan) is the only approved HIV ICA test in Japan that can detect anti-HIV-1/2 antibodies (Abs) and free HIV-1 antigen (Ag) separately. Other HIV *in vitro* diagnostics (IVDs) for screening test that are approved in Japan include HIV Ag/Ab combination tests, with the exception of Genedia HIV-1/2 mix PA (Fujirebio, Tokyo, Japan). HIV screening test-positive cases are examined using the HIV Ab confirmatory test with the diagnostic test algorithm in Japan. Most specimens that are interpreted as positive in the screening test based on HIV-1 Ag detection may be considered negative or indeterminate when tested using HIV Ab confirmatory test.

One Ag-positive sample was tested using Determine HIV Early Detect in our post-marketing surveillance study using 106 HIV-negative plasma specimens [2]. Mourez et al. described one Ag-positive case among 200 HIV-negative samples that were identified using Determine HIV Early Detect [3]. Diagnostic algorithms should be developed to exclude HIV-1 Ag false-positive cases.

In such cases, HIV-1 nucleotide amplification test (NAT) is effective as HIV-1 confirmatory test. The performance of IVDs in amplifying HIV-1 RNA has recently improved dramatically, and highly sensitive tests using highly controlled systems can be performed. Qualitative NATs that can be used to diagnose HIV infection and differentiate between HIV-1 and HIV-2 have recently emerged. However, these IVDs require expensive equipment and specialized reagents, making them difficult to introduce into areas with low HIV prevalence and small- and medium-sized diagnostic laboratories. In addition, no IVDs for qualitative NATs have been approved in Japan.

In this study, we investigated a simple and inexpensive in-house qualitative HIV-1 NAT (HIV-1 QL-NAT) as an adjunct HIV-1 confirmatory test for specimens that were HIV Ag-positive on HIV screening test and indeterminate or negative using an HIV Ab confirmatory test.

## 2 Materials and methods

### 2.1 RNA extraction

All samples used in this study were plasma. They were thawed, aliquoted, and stored at −80 °C until use. New tubes were thawed and used for each test. RNA was extracted from plasma samples (150 μL) and eluted with 50 μL of RNase-free water using NucleoSpin RNA Virus (Macherey-Nagel GmbH, Düren, Nordrhein-Westfalen, Germany). Then, 2.5 μL of the eluate was used for amplification.

### 2.2 HIV-1 QL-NAT

The HIV-1 QL-NAT was performed using two primer pairs (QL1 and QL2). The following primers were used: forward primer (580A) (5ʹ-GAT GGG TGC GAG AGC GTC-3ʹ [789–806 HXB2]) and reverse primer (612B) (5ʹ-GCT CCC TGC TTG CCC ATA CTA-3ʹ [910-890 HXB2]) for QL1; forward primer (Gag183UF) (5ʹ-CTA GCA GTG GCG CCC GAA CAG-3ʹ [629–649 HXB2]) and reverse primer (Gag187LR) (5ʹ-CCA TCT CTC TCC TTC TAG CCT CCG CTA GTC A-3ʹ [793-763 HXB2]) for QL2. The primers for QL2 were the same as those used in the TaqMan PCR method described by Kaur et al. [4]. GoTaq 1-Step RT-qPCR System (Promega, Madison, WI, USA) was used for analysis with the StepOne Plus Real-Time PCR System (Applied Biosystems Inc., Foster City, CA, USA). Electrophoresis was performed using 3 % Prime Gel Agarose PCR-Sieve HRS (Takara Bio Inc., Shiga, Japan), and the bands were observed after staining with Gel Red (Cosmobio Inc., Tokyo, Japan). Details, including the fragment size and reaction cycle, are summarized in Supplementary Table 1. A representative test is shown in Supplementary Fig. 1. In some tests, the same RNA was analyzed with two TaqMan PCR methods [4,5] using TaqMan RNA-to-Ct 1-Step Kit (Applied Biosystems), according to the manufacturer's instruction.

### 2.3 Positive/negative controls for HIV-1 QL-NAT

Culture supernatant from HIV-1_LAI_-infected MT2 cells was used to prepare the in-house HIV-1-positive standard. The supernatant was incubated at 60 °C for 1 h, and the inactivation was confirmed using the following criteria after MAGIC5 cell infection: 1) no cytopathic effect observed and 2) no blue-stained cells observed after β-galactosidase staining [6]. The inactivated supernatant was added to Base Matrix 53 (BM53; SeraCare Life Sciences, Milford, MA, USA) and named 18-00. The HIV-1 RNA was quantified three times using cobas HIV-1 Quantitative (Roche Molecular Systems, South Branchburg, NJ, USA), and the geometric mean of 3,890,000 copies (cp)/mL was used as the copy number. To determine the limit of detection (LOD), serial dilution of 18-00 using BM53 was examined three times (Table 1). Five times the LOD concentration was prepared using BM53, dispensed, and used as a positive control in the HIV-1 QL-NAT. BM53 was used as the negative control. RNA from these controls was extracted for all tests to confirm whether the RNA extraction kit was working.

**Table 1:** Limit of detection for QL1 and QL2.

Dilution	QL1									QL2								
	Exp. 1			Exp. 2			Exp. 3			Exp. 1			Exp. 2			Exp. 3		
	Ct^†^	Tm^\^	EP^#^	Ct	Tm	EP	Ct	Tm	EP	Ct	Tm	EP	Ct	Tm	EP	Ct	Tm	EP
10^–2^	27.56	79.82	+	27.58	80.28	+	27.25	79.82	+	28.40	86.82	+	28.36	87.32	+	28.23	87.33	+
10^–2.5^	28.37	79.82	+	29.32	80.28	+	28.31	79.82	+	30.43	86.82	+	29.77	87.32	+	29.85	87.33	+
10^–3^	31.73	79.82	+	32.26	80.28	+	30.92	79.82	+	30.82	86.82	+	31.77	87.32	+	31.49	87.33	+
10^–3.5^	32.79	80.33	+	32.64	80.28	+	31.87	79.82	+	31.74	86.82	+	33.31	87.32	+	33.35	87.33	+
10^–4^	35.21	80.33	+	33.18	80.28	+	32.93	79.82	+	UD	62.30	–	33.47	87.32	+	UD	61.84	–
10^–4.5^	UD^¶^	61.81	–	UD	62.84	–	UD	61.85	–	UD	62.30	–	UD	60.68	–	UD	61.84	–
10^–5^	UD	61.81	–	UD	62.84	–	UD	61.85	–	UD	61.24	–	UD	60.68	–	UD	61.84	–
BM53^§^	UD	62.83	–	UD	60.70	–	UD	61.84	–	UD	61.24	–	UD	60.68	–	UD	61.84	–

Ct^†^, Threshold cycle; Tm^\^, Melting temperature; EP^#^, agarose gel electrophoresis; BM53^§^, BaseMatrix53; UD^¶^, undetermined.

### 2.4 Commercial HIV-1 performance/seroconversion panels

ACCURUN315 series 100, 200, 300, 400, and 500 (Boston Biomedica Inc., Bridgewater, MA, USA), Antigen Mixed Titer Performance Panels PRA201 and PRA202 (Boston Biomedica Inc.), AccuSet HIV-1 Early Infection Performance Panel 0800-0394 (SeraCare Life Sciences), and 15 seroconversion panels (PRB905, PRB910, PRB912, PRB914, PRB916, PRB919, PRB921, PRB922, PRB923, PRB924, PRB925, PRB971, PRB973, PRB976, and PRB977; SeraCare Life Sciences) were used in this study.

### 2.5 HIV-1 subtypes/circulating recombinant forms/groups Ag panel

The in-house HIV-1 Ag panel, including 11 HIV-1 subtypes, circulating recombinant forms (CRFs), and groups (HIV-1 types) [7], was diluted with BM53 and examined.

### 2.6 HIV-negative clinical specimens

HIV-1-negative plasma specimens were assessed as previously described [2]. Specimens from blood donors who were ineligible for transfusion were provided by the Japanese Red Cross Blood Center in accordance with an application for the use of donated blood in Japan, based on the guidelines for the use of donated blood in research and development. Specimen information was anonymized, and a decoding index was not created. Ethics approval was obtained from the Ethical Committee of the National Institute of Infectious Diseases (No. 1082).

### 2.7 Statistical analysis

Dissociation analysis of melting temperature (Tm) for HIV-1-positive and -negative samples was performed using a method that was selected based on the results of the F test for homogeneity of variance. All analyses were performed using GraphPad Prism 9 software (GraphPad Software, LLC, San Diego, CA, USA).

### 2.8 HIV serodiagnosis

Determine HIV Early Detect, Genscreen ULTRA HIV Ag-Ab (Bio-Rad Laboratories, Hercules, CA, USA), and Geenius HIV-1/2 Confirmatory Assay (Bio-Rad Laboratories) were used for serodiagnosis.

## 3 Results

### 3.1 Determining the LOD of the HIV-1 QL-NAT

Serial dilution of 18-00 using BM53 was examined three times. QL1 was detected at a dilution of 10^−4^ in all three tests (Table 1). Two of the three tests detected QL2 at a dilution of 10^−3.5^ and at 10^−4^ in one test (Table 1). The results were the same using fluorescent signal detection and agarose gel electrophoresis (Table 1). The LOD for QL1 was 389 cp/mL and the geometric mean, 838 cp/mL, was used as the LOD for QL2.

In the evaluation using ACCURUN315, 315–500, 400, 300, and 200, but not 315-100, were detected using both methods (Supplementary Table 2). The results were the same using fluorescent signal detection and agarose gel electrophoresis. The copy number for 315-200 was 820 cp/mL (Supplementary Table 2).

### 3.2 Evaluation of HIV-1-type specificity for HIV-1 QL-NAT

The in-house diluted HIV-1 Ag panel was examined (Table 2). Although most results obtained using fluorescent signal detection and agarose gel electrophoresis were consistent between QL1 and QL2, a fluorescent signal was detected, however, no specific band was observed in the first experiment for 100 cp/mL of 00 KE_KNH1144 using QL1 (Table 2, Supplementary F. 2A). Moreover, both the fluorescent signal and a specific band were detected, although the Tm values were close to 60 in five samples (93RW_024_Exp. 1–300 cp/mL, 02 ET_288_Exp. 2–100 cp/mL, 93UG_065_Exp. 2–1000 cp/mL, 98 TH_NP1251_Exp. 1–100 cp/mL, and 00CN_HH040_Exp. 1–300 cp/mL) using QL2 (Table 2, Supplementary F. 2B–2F).

**Table 2:** Evaluation of HIV-1-type specificity using QL1 and QL2.

Sample	HIV--1 type	RNA copies	QL1				QL2				Kondo et al. [5]		Kaur et al. [4]	
			Exp. 1		Exp. 2		Exp. 1		Exp. 2		Exp. 1	Exp. 2	Exp. 1	Exp. 2
		cp/mL	Ct^†^	Tm^\^	Ct	Tm	Ct	Tm	Ct	Tm	Ct	Ct	Ct	Ct
93RW_024	Subtype A	3000	30.52	80.79	31.63	79.79	32.74	87.32	34.28	87.30	36.19	37.42	33.96	34.43
		1000	33.78	81.29	33.26	79.79	35.65	86.80	37.54	86.77	38.33	39.28	36.08	37.01
		300	35.51	81.29	33.60	79.79	36.48	86.80	38.07	64.27	UD	UD	36.80	39.16
		100	UD^¶^	61.80	35.40	80.78	37.69	86.80	UD	61.80	UD	UD	UD	39.05
00 KE_KNH1144		3000	31.21	79.31	31.46	78.79	33.42	87.29	33.43	87.27	36.19	37.29	35.48	35.08
		1000	32.97	79.31	33.20	78.79	36.20	87.29	37.61	87.27	38.01	UD	37.02	36.73
		300	33.74	79.31	35.31	79.29	37.34	87.29	37.96	86.29	39.52	UD	38.33	38.13
		100	37.22	78.28	UD	61.77	UD	62.29	UD	61.81	UD	UD	UD	UD
00 KE_KNH1207		3000	32.93	80.31	30.69	79.80	34.01	87.31	33.88	86.80	37.20	38.12	35.28	36.16
		1000	36.29	80.81	34.40	79.80	36.21	87.31	35.97	86.80	39.30	39.28	37.86	37.09
		300	36.66	79.80	UD	62.33	UD	61.83	37.42	86.80	UD	UD	38.87	UD
		100	UD	62.35	UD	62.33	UD	61.83	UD	61.81	UD	UD	39.00	UD
94US_33931 N	Subtype B	3000	30.35	80.29	29.87	79.79	32.68	86.80	33.18	85.73	34.46	34.90	33.80	34.22
		1000	32.79	79.80	33.70	80.28	34.84	87.32	34.81	86.77	37.38	38.16	36.41	36.11
		300	34.41	79.80	35.00	79.79	36.63	86.80	36.72	86.77	39.80	39.22	37.24	37.95
		100	34.64	80.79	UD	61.27	UD	62.31	UD	61.80	UD	UD	38.46	UD
84US_MNp		3000	31.89	80.30	32.12	79.80	34.83	87.77	35.68	87.27	36.48	36.62	36.19	35.64
		1000	33.79	80.30	33.42	79.80	35.74	87.77	UD	62.28	38.19	37.80	38.81	37.24
		300	UD	61.78	33.65	79.80	UD	62.29	37.16	87.27	UD	UD	37.80	37.35
		100	UD	61.25	UD	61.77	UD	60.77	UD	61.81	UD	UD	UD	UD
96 TH_NP1538		3000	30.88	79.80	30.29	79.80	32.12	87.31	32.93	86.80	36.67	37.06	34.15	34.14
		1000	34.39	80.31	33.46	79.80	34.42	87.31	35.37	86.80	38.49	37.27	37.45	36.31
		300	32.82	79.80	33.72	79.80	35.29	87.31	36.29	86.80	39.44	39.10	37.83	37.54
		100	34.98	79.80	UD	62.33	UD	61.29	UD	60.72	UD	UD	UD	38.60
02 ET_288	Subtype C	3000	30.24	80.30	30.37	78.80	33.27	86.81	33.80	86.77	35.84	36.63	33.77	34.12
		1000	31.76	79.81	32.45	79.79	35.19	86.81	34.96	86.77	37.81	37.89	36.47	35.98
		300	33.89	79.81	35.30	79.30	37.43	86.81	37.58	86.77	UD	38.23	36.61	37.47
		100	35.02	79.32	35.16	78.80	UD	62.32	38.99	63.31	UD	UD	37.79	UD
93IN101		3000	30.00	79.31	31.21	78.79	31.21	85.82	31.64	85.30	34.85	34.74	33.12	32.78
		1000	32.79	78.81	33.69	78.79	34.11	86.31	33.96	85.30	36.42	35.74	35.70	34.90
		300	36.21	79.82	33.17	78.79	35.16	86.31	35.68	85.30	38.61	38.12	36.14	36.14
		100	UD	61.79	35.29	79.29	38.64	84.81	37.77	85.30	UD	UD	39.06	38.91
96USNG31		3000	30.74	78.26	30.71	78.24	32.81	85.82	33.68	85.81	37.19	37.07	35.22	34.38
		1000	32.67	78.26	32.51	77.71	34.19	86.32	35.40	85.81	38.64	39.33	35.54	35.85
		300	32.95	78.26	34.39	77.71	36.34	85.82	37.64	85.81	UD	UD	37.45	38.83
		100	UD	61.84	UD	60.69	UD	61.29	UD	60.72	UD	UD	UD	UD

Sample	HIV--1 type	RNA copies	QL1				QL2				Kondo et al. [5]		Kaur et al. [4]	
			Exp. 1		Exp. 2		Exp. 1		Exp. 2		Exp. 1	Exp. 2	Exp. 1	Exp. 2
		cp/mL	Ct^†^	Tm^\^	Ct	Tm	Ct	Tm	Ct	Tm	Ct	Ct	Ct	Ct
93UG_065	Subtype D	3000	31.64	79.81	31.54	80.28	34.95	86.81	33.66	86.77	36.05	35.84	34.81	34.52
		1000	32.66	81.30	32.93	79.79	37.94	86.81	38.21	62.81	38.13	37.74	36.81	36.39
		300	UD	61.81	34.59	79.79	37.72	86.81	UD	61.80	UD	UD	39.23	37.77
		100	UD	62.31	34.21	80.28	36.49	87.32	UD	61.80	UD	UD	39.99	UD
00 KE_NKU3006		3000	30.91	78.81	31.45	79.29	33.70	87.30	33.86	85.80	36.00	36.91	36.22	35.57
		1000	33.20	78.81	32.77	79.29	35.88	86.81	35.61	85.80	38.93	UD	35.53	36.15
		300	34.00	78.81	34.68	78.79	35.69	86.81	38.39	86.81	39.85	UD	37.36	UD
		100	35.30	79.82	36.39	78.79	UD	62.30	UD	61.81	UD	UD	UD	UD
BZ163	Subtype F	3000	29.70	82.29	29.83	81.28	31.92	87.32	32.58	85.75	34.19	34.90	33.54	32.88
		1000	31.98	82.29	32.32	81.28	33.50	86.81	35.32	86.26	37.78	36.94	34.84	35.50
		300	33.12	82.29	33.81	81.28	36.43	86.81	35.39	85.75	38.43	37.68	37.36	36.79
		100	34.31	82.29	35.19	81.28	37.62	86.81	37.88	85.75	UD	UD	UD	UD
BCI-R07		3000	30.45	79.82	30.35	79.81	33.52	85.82	33.39	86.31	35.44	35.68	33.98	34.32
		1000	32.88	79.82	32.02	79.81	37.52	85.82	35.54	86.31	37.31	38.20	36.83	35.78
		300	33.24	79.82	33.77	80.29	36.39	86.31	37.47	85.81	37.85	38.52	38.27	37.27
		100	35.28	79.82	UD	60.73	UD	62.30	UD	60.78	UD	UD	38.98	UD
BCF-DIOUM	Subtype G	3000	30.55	79.80	30.40	79.80	32.36	84.81	33.11	84.78	36.26	36.03	33.89	34.75
		1000	32.21	79.80	32.77	79.80	34.27	84.81	35.10	84.78	37.09	38.00	35.37	35.60
		300	32.53	79.80	33.64	79.80	35.71	85.82	UD	60.72	UD	38.94	37.36	37.53
		100	UD	60.71	34.98	79.80	37.35	86.32	UD	60.72	UD	UD	UD	37.81
96 TH_M02138	CRF01_AE	3000	31.23	79.81	31.26	79.80	33.28	84.81	34.39	84.80	36.53	35.75	34.11	33.94
		1000	33.45	80.30	32.94	79.80	34.89	84.81	34.85	84.80	36.76	38.29	38.77	36.20
		300	34.52	80.30	33.80	79.80	35.71	84.81	UD	61.81	UD	38.61	39.75	36.97
		100	UD	61.81	UD	62.30	UD	62.32	UD	61.81	UD	UD	UD	UD
90 TH_CM244		3000	31.61	79.82	32.69	79.81	33.41	84.81	33.20	85.31	36.06	36.05	34.44	34.19
		1000	33.37	79.82	33.22	79.81	37.28	84.81	36.87	84.81	37.72	38.04	35.47	35.49
		300	UD	61.79	35.29	80.29	38.23	86.31	36.77	84.81	39.26	39.81	38.99	39.32
		100	UD	61.79	UD	61.79	37.66	86.31	UD	61.82	UD	UD	38.82	UD
98 TH_NP1251		3000	30.66	79.79	30.45	79.30	32.96	85.81	33.25	85.31	36.01	37.22	34.69	34.25
		1000	33.40	79.79	34.48	79.81	33.92	86.31	34.59	85.31	37.64	38.10	35.94	36.57
		300	34.33	79.79	33.01	79.30	37.75	85.81	37.56	85.31	38.18	38.81	37.16	37.91
		100	UD	63.34	UD	63.34	38.01	63.83	UD	61.28	UD	UD	38.74	UD
01CM_0005BBY	CRF02_AG	3000	30.88	80.29	31.48	80.29	33.97	85.73	33.76	85.27	36.07	35.99	34.33	34.43
		1000	32.99	80.29	33.84	79.80	38.39	85.73	36.94	84.80	39.28	37.86	35.39	36.81
		300	34.76	80.29	36.30	80.29	36.46	85.73	37.79	84.80	UD	39.08	36.88	37.92
		100	35.23	80.78	UD	61.80	UD	60.56	37.83	84.80	UD	UD	39.17	UD

Sample	HIV--1 type	RNA copies	QL1				QL2				Kondo et al. [5]		Kaur et al. [4]	
			Exp. 1		Exp. 2		Exp. 1		Exp. 2		Exp. 1	Exp. 2	Exp. 1	Exp. 2
		cp/mL	Ct^†^	Tm^\^	Ct	Tm	Ct	Tm	Ct	Tm	Ct	Ct	Ct	Ct
02CM_1970LE	CRF02_AG	3000	30.63	78.79	30.61	78.80	31.68	85.31	32.78	84.81	35.34	35.56	33.26	33.18
		1000	33.88	78.79	32.64	78.80	33.77	85.31	34.51	85.31	38.53	37.37	35.61	35.92
		300	32.55	78.79	34.18	78.80	36.24	85.31	38.09	84.81	37.75	38.84	37.03	36.99
		100	34.42	78.79	UD	61.79	UD	61.82	UD	60.78	UD	UD	37.71	39.12
91DJ_263		3000	31.26	78.77	31.87	79.30	32.10	85.81	32.48	86.32	36.07	37.29	33.43	34.17
		1000	32.09	79.79	31.97	79.30	34.18	86.31	36.22	86.32	39.30	38.40	35.72	36.60
		300	35.21	79.79	33.28	79.81	35.87	85.81	35.12	86.32	39.51	UD	36.13	35.90
		100	35.54	79.79	35.29	79.81	37.95	85.81	36.00	86.32	UD	UD	UD	UD
99CN013	CRF07_BC	3000	30.61	79.30	30.70	79.31	32.41	85.73	32.32	85.75	36.07	35.66	33.45	33.15
		1000	32.95	78.81	32.60	78.81	34.38	85.73	35.90	85.75	39.55	37.82	35.80	35.36
		300	33.79	80.29	33.61	78.81	37.11	85.73	35.71	85.75	38.84	38.50	37.97	36.46
		100	UD	60.74	UD	60.75	36.59	85.73	38.55	86.26	UD	UD	UD	39.42
00CN_HH043		3000	30.25	77.74	30.69	78.28	31.68	85.31	32.31	84.81	36.89	36.63	32.99	33.16
		1000	32.43	78.79	31.86	78.28	33.93	85.81	35.46	86.31	39.54	39.56	34.95	34.81
		300	32.38	78.79	33.27	78.80	35.24	85.81	34.70	85.81	UD	39.49	36.47	36.28
		100	34.31	78.79	UD	61.79	UD	61.82	UD	60.78	UD	UD	37.98	39.19
00CN_HH040	CRF08_BC	3000	30.94	78.81	30.89	78.81	32.23	85.73	32.23	86.25	34.51	34.64	32.94	33.00
		1000	32.98	79.30	34.89	78.81	35.91	85.73	35.44	86.25	36.60	36.82	34.87	35.22
		300	33.52	79.30	33.67	78.81	38.43	64.27	35.66	86.25	37.17	37.67	36.55	35.32
		100	35.38	78.81	34.22	78.81	37.66	85.73	36.73	85.73	UD	UD	37.79	UD
00CN_DL001		3000	29.42	78.79	29.76	78.81	31.41	85.81	31.84	84.81	34.96	34.92	32.74	32.35
		1000	30.89	78.79	31.16	78.81	34.18	85.81	33.98	84.81	36.36	36.10	34.34	34.89
		300	32.70	78.79	31.81	78.81	34.74	85.81	37.41	85.82	39.15	37.70	35.63	35.93
		100	UD	61.78	34.84	78.81	UD	61.29	36.61	85.82	38.77	39.24	37.88	38.46
00CN_QJ001		3000	30.66	78.77	30.29	79.30	31.53	86.31	31.60	86.32	35.89	35.57	33.51	33.31
		1000	33.27	79.28	35.02	79.30	34.87	85.81	34.94	86.32	37.16	36.74	35.36	35.86
		300	35.47	79.28	UD	63.34	36.43	86.31	36.43	86.32	39.01	39.35	36.69	37.20
		100	UD	61.25	UD	63.34	UD	62.32	36.12	86.32	UD	UD	UD	UD
BCF06	Group O	3000	29.61	80.29	29.68	79.80	UD	61.80	UD	64.27	UD	UD	UD	UD
		1000	31.38	80.29	33.35	80.29	UD	61.80	UD	64.27	UD	UD	UD	UD
		300	32.84	80.29	35.19	79.80	UD	61.80	UD	61.81	UD	UD	UD	UD
		100	35.22	80.29	UD	60.74	UD	61.80	UD	61.81	UD	UD	UD	UD
I–2478B		3000	28.85	79.80	28.95	78.81	UD	61.82	UD	61.29	UD	UD	UD	UD
		1000	31.18	79.80	31.28	78.81	UD	61.82	UD	62.28	UD	UD	UD	UD
		300	31.89	79.80	32.88	78.81	UD	61.29	UD	62.28	UD	UD	UD	UD
		100	33.55	79.80	34.56	78.81	UD	61.29	UD	62.28	UD	UD	UD	UD

Ct^†^, Threshold cycle; Tm^\^, Melting temperature; UD^¶^, undetermined.

Two HIV-1 group O samples were detected using QL1, although detection failed using QL2 (Table 2). Twenty-five other samples showed positive results using both methods, up to at least 1000 cp/mL. The specific signal was observed using two TaqMan PCR methods [4,5] (Table 2). The detection sensitivity was nearly the same as that of QL1 and QL2, although two group O samples could not be detected (Table 2).

### 3.3 Interpretation of QL1 and QL2 results

**Fig. 1:**
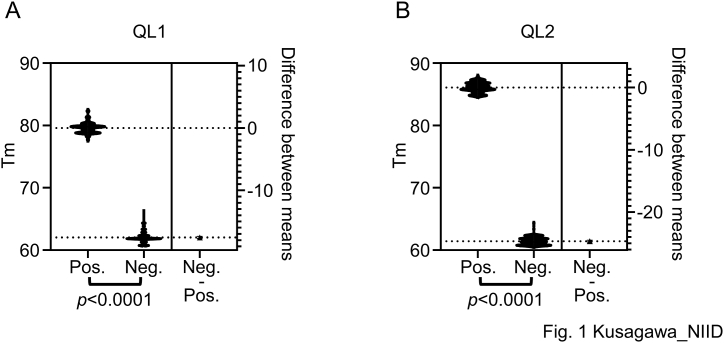
Dissociation analysis of Tm results from in-house qualitative HIV-1 nucleotide amplification tests QL1 (A) and QL2 (B).

**Fig. 2:**
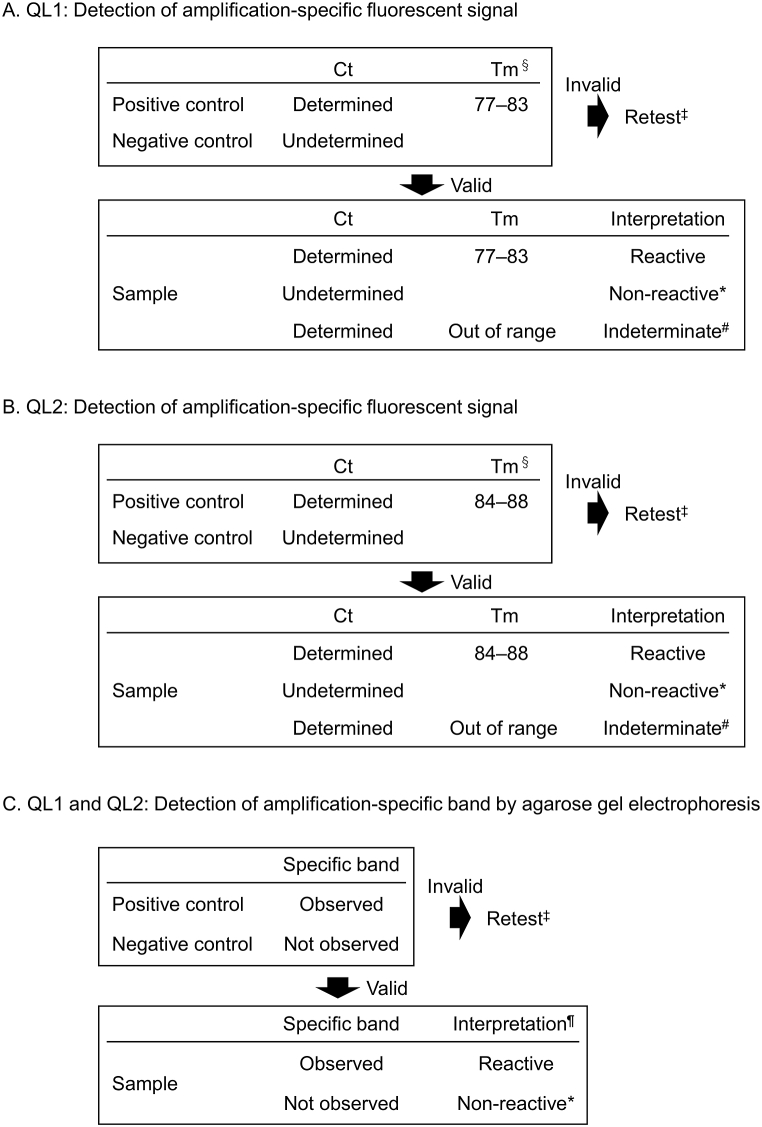
Interpretation of results for QL1 and QL2. Tm§, depending on the equipment; Retest‡, retest from RNA extraction for invalid cases; Non-reactive*, not equal to HIV-1-uninfected; Indeterminate#, retest or examine using agarose gel electrophoresis; Interpretation¶, if the specific band of the sample was thinner than that of the positive control, or if bands were observed at an unexpected position except for non-reacted primer, the result was considered indeterminate.

In our previous study, 106 HIV-negative plasma specimens, including one HIV-1 Ag false-positive case (Ba01), were examined and found to be HIV-1-negative [2]. The distribution of Tm values in the positive and negative results using the in-house HIV-1 Ag panel (Table 2) and 106 HIV-negative plasma specimens [2] are shown in Fig. 1. Five tests in which the fluorescent signal was detected and the Tm value was close to 60 were excluded from the analysis (Table 2). The Tm values of positive samples ranged from 77.71 to 82.29 (n = 182; average, 79.60) for QL1 (Fig. 1A) and from 84.78 to 87.77 (n = 158; average, 86.09) for QL2 (Fig. 1B). The Tm results of the negative samples ranged from 60.09 to 66.30 (n = 260; average, 62.00) for QL1 (Figs. 1A) and 60.56–64.27 (n = 279; average, 61.42) for QL2 (Fig. 1B). The Mann–Whitney *U* test was used to examine the distribution of Tm values among positive and negative samples because the Tm values showed unequal variance using the F test for both methods. The distribution of Tm values between HIV-positive and HIV-negative samples differed significantly (*p* < 0.0001; Fig. 1A and B). The interpretation criteria for fluorescent signal detection are described in Fig. 2A and B based on these data. Interpretation using agarose gel electrophoresis was determined by the presence or absence of a band with a specific size (Supplementary Tab. 1, Supplementary F. 1 and F. 2C).

### 3.4 Evaluation of HIV-1 QL-NAT using HIV-1 Ag-positive samples in the ICA test

Commercial HIV-1 seroconversion/performance panels were examined using Determine HIV Early Detect. An HIV-1 Ag band was observed in 27 samples (Table 3). All samples were positive, although the HIV-1 Ag-pseudo-positive sample (Ba01) [2] was negative in the supplemental HIV screening test using Genscreen ULTRA HIV Ag-Ab (Table 3). Four HIV-1-indeterminate, two HIV-2-indeterminate, one HIV-indeterminate, and 20 HIV-negative samples were included in the HIV-Ab confirmatory test using the Geenius HIV-1/2 confirmatory assay (Table 3). No fluorescent signals were observed using the HIV-2 NAT [8] (Supplementary Fig. 3), and no specific bands were observed using NEW LAV BLOT II (Bio-Rad Laboratories, Supplementary Fig. 3) in HIV-2-indeterminate (PRB905-08 and PRB971-03) and HIV-indeterminate (PRB971-04) samples. All 28 samples were examined using QL1, QL2, and the two TaqMan PCR methods [4,5]. Twenty-seven of the 28 samples were reactive, although Ba01 was non-reactive using all four methods (Table 3). These results are consistent with those of the agarose gel electrophoresis using QL1 and QL2 (Table 3). Ten of the 27 samples were analyzed together using the HIV-1 RNA quantification standard in the two TaqMan PCR tests. The HIV-1 RNA copy number was ≥180,000 cp/mL (Table 3).

**Table 3:** Evaluation of in-house qualitative HIV-1 nucleotide amplification tests in Ag-positive cases using Determine HIV Early Detect and Geenius HIV 1/2 Confirmatory Assay-negative or -indeterminate samples.

ID	Determine HIV Early Detect			Genscreen ULTRA HIV Ag-Ab"		Geenius HIV 1/2 Confirmatory Assay								HIV-1 NAT											
														QL1				QL2				Kondo et al. [5]		Kaur et al. [4]	
						HIV-2		HIV-1				CTRL^¶^	Result	Exp. 1		Exp. 2		Exp. 1		Exp. 2		Exp. 1	Exp. 2	Exp. 1	Exp. 2
	IC^$^	Ag	Ab	C.O.I.	Result	gp36	gp140	p31	gp160	p24	gp41			Sig*	EP#	Sig	EP#	Sig	EP#	Sig	EP#	Sig	Sig	Sig	Sig
<Seroconversion Panels>																									
PRB905_08	+	+	–	4.11	Pos.	–	±	–	–	–	–	+	HIV-2 Ind^‡^	+	+	+	+	+	+	+	+	+	+	+	+
PRB905_09	+	+	+	>12.56	Pos.	–	–	–	–	–	–	+	Neg.	+	+	+	+	+	+	+	+	+	+	+	+
PRB910_02	+	+	–	7.11	Pos.	–	–	–	–	–	–	+	Neg.	+	+	+	+	+	+	+	+	+	+	+	+
PRB912_01	+	+	+	10.47	Pos.	–	–	–	–	–	–	+	Neg.	+	+	+	+	+	+	+	+	+	+	+	+
PRB916_04	+	+	–	11.99	Pos.	–	–	–	–	–	–	+	Neg.	+	+	+	+	+	+	+	+	+	+	+	+
PRB919_01	+	+	–	5.32	Pos.	–	–	–	–	–	–	+	Neg.	+	+	+	+	+	+	+	+	+	+	+	+
PRB921_01	+	+	–	>12.56	Pos.	–	–	–	–	–	–	+	Neg.	+	+	+	+	+	+	+	+	+	+	+	+
PRB921_02	+	+	+	>12.56	Pos.	–	–	–	–	–	+	+	HIV-1 Ind	+	+	+	+	+	+	+	+	+	+	+	+
PRB922_01	+	+	–	10.16	Pos.	–	–	–	–	–	–	+	Neg.	+	+	+	+	+	+	+	+	+	+	+	+
PRB922_02	+	+	+	11.32	Pos.	–	–	–	–	–	+	+	HIV-1 Ind	+	+	+	+	+	+	+	+	+	+	+	+
PRB923_09	+	+	–	12.24	Pos.	–	–	–	–	–	–	+	Neg.	+	+	+	+	+	+	+	+	+	+	+	+
PRB924_05	+	+	–	10.22	Pos.	–	–	–	–	–	–	+	Neg.	+	+	+	+	+	+	+	+	+	+	+	+
PRB924_06	+	+	+	11.17	Pos.	–	–	–	–	–	–	+	Neg.	+	+	+	+	+	+	+	+	+	+	+	+
PRB925_05	+	+	+	12.25	Pos.	–	–	–	–	–	±	+	HIV-1 Ind	+	+	+	+	+	+	+	+	+	+	+	+
PRB971_03	+	+	–	>12.43	Pos.	–	+	–	–	–	–	+	HIV-2 Ind	+	+	+	+	+	+	+	+	714,000	489,000	781,000	852,000
PRB971_04	+	+	+	>12.43	Pos.	–	+	–	–	–	+	+	HIV Ind	+	+	+	+	+	+	+	+	3,820,000	3,190,000	4,890,000	3,990,000
PRB973_04	+	+	+	8.62	Pos.	–	–	–	–	–	–	+	Neg.	+	+	+	+	+	+	+	+	1,330,000	1,270,000	1,730,000	1,860,000
PRB976_03	+	+	–	10.17	Pos.	–	–	–	–	–	–	+	Neg.	+	+	+	+	+	+	+	+	650,000	670,000	1,300,000	1,300,000
PRB976_04	+	+	–	>12.43	Pos.	–	–	–	–	–	–	+	Neg.	+	+	+	+	+	+	+	+	1,300,000	1,900,000	4,000,000	3,800,000
PRB977_03	+	+	+	>12.43	Pos.	–	–	–	–	–	–	+	Neg.	+	+	+	+	+	+	+	+	2,300,000	1,700,000	2,500,000	2,700,000
<HIV-1 Antigen Performance Panels>																									
PRA201_05	+	+	–	8.55	Pos.	–	–	–	–	–	–	+	Neg.	+	+	+	+	+	+	+	+	+	+	+	+
PRA201_17	+	+	–	4.36	Pos.	–	–	–	–	–	–	+	Neg.	+	+	+	+	+	+	+	+	+	+	+	+
PRA201_18	+	+	+	>12.38	Pos.	–	–	–	–	–	–	+	Neg.	+	+	+	+	+	+	+	+	+	+	+	+
PRA202_12	+	+	+	10.66	Pos.	–	–	–	–	–	+	+	HIV-1 Ind	+	+	+	+	+	+	+	+	480,000	580,000	390,000	380,000
PRA202_19	+	+	+	>12.38	Pos.	–	–	–	–	–	–	+	Neg.	+	+	+	+	+	+	+	+	2,100,000	2,600,000	4,200,000	4,900,000
<HIV-1 Early Infection Performance Panel>																									
0800_0394_13	+	+	–	5.27	Pos.	–	–	–	–	–	–	+	Neg.	+	+	+	+	+	+	+	+	180,000	250,000	340,000	320,000
0800_0394_15	+	+	–	8.51	Pos.	–	–	–	–	–	–	+	Neg.	+	+	+	+	+	+	+	+	580,000	1,200,000	1,100,000	960,000
<HIV-Negative clinical specimen>																									
Ba01	+	+	–	0.381	Neg.	–	–	–	–	–	–	+	Neg.	–	–	–	–	–	–	–	–	–	–	–	–

IC^$^, internal control; CTRL^¶^, control; Sig*, fluorescent signal detection; EP^#^, agarose gel electrophoresis; Ind.^‡^, indeterminate.

## 4 Discussion

In this study, we examined a simple and inexpensive HIV-1 QL-NAT as an adjunct HIV-1 confirmatory test. Real-time PCR using the intercalator method and conventional RT-PCR using two primer pairs were chosen as the HIV-1 QL-NATs based on their purchase costs and validation and maintenance requirements.

TaqMan method detects the target sequence using a primer pair and TaqMan probe; therefore, the target sequence can be detected more specifically. However, TaqMan probes are expensive, and their shelf life is limited. Preparation of each test-specific TaqMan probe is challenging for small- and medium-sized diagnostic laboratories. HIV-1 QL-NATs can share reagents and equipment, except primers, with multiple infectious diagnoses, and are useful as adjunct HIV confirmatory test. Laboratories without real-time PCR equipment can perform amplification reactions using conventional RT-PCR reagents and confirm the results using agarose gel electrophoresis. The analytical sensitivities of the two detection methods were almost equal.

The LOD for QL1 and QL2, using the in-house HIV-1 standard and ACCURUN315, was estimated to be < 1000 cp/mL. We reported that the LOD for HIV-1 Ag detection using Determine HIV Early Detect ranged from 100,000 to 1,000,000 cp/mL using in-house HIV-1 Ag panel [7]. The detection sensitivity of IVDs for HIV screening tests based on chemiluminescence or enzyme immunoreaction detection is estimated to be 10–100-times higher than that of Determine HIV Early Detect [7]. The analytical sensitivities of QL1 and QL2 were sufficient as HIV-1 confirmatory test for HIV-1 Ag-positive cases.

We examined HIV-1-type specificity using our HIV-1 Ag panel dilutions [7]. All HIV-1 subtype/CRF samples showed positive results of up to 1000 cp/mL for both QL1 and QL2. No variations in detection sensitivity according to HIV-1 subtype/CRF were observed. QL1, but not QL2, detected two HIV-1 group O samples with similar sensitivities. Approximately 80 % of HIV-1 cases in Japan are of subtype B, although various subtypes/CRFs were recently detected [9,10]. However, no HIV-1 group O infections have been reported to date. Both QL1 and QL2 can be used in areas with similar HIV-1 epidemic situations as in Japan.

Although we did not encounter non-specific amplification cases with QL1 and QL2, there is always a risk of false-positive results due to cross-contamination during nucleic acid amplification testing. Laboratory setup is important when using the HIV-1 QL-NAT. Preparing a positive control containing a concentration near the LOD for HIV-1 and a negative control using HIV-negative plasma was also necessary. Positive and negative controls were examined using sample testing to confirm that the test was performed correctly. We set the Tm value criteria for interpreting the results because there is a risk that the intercalator method may detect primer dimers or non-specific PCR products. The precision of the test may also be improved if PCR products are subjected to periodic electrophoresis.

The HIV-1 QL-NAT was performed for Ag-positive cases, which were determined using Determine Early Detect. All HIV-1 Ag-positive cases were determined to be reactive, and one HIV-1 Ag false-positive case was excluded. The same result was obtained in the supplemental HIV screening test using Genscreen ULTRA HIV Ag-Ab. Ten of 27 samples were quantified using two TaqMan PCR methods [4,5], and the HIV-1 RNA copy numbers were 180,000 cp/mL or higher. In the analysis of Ag-positive clinical specimens using Determine HIV Early Detect, the HIV-1 copy number distribution ranged from 533,000 to 65,700,000 cp/mL (n = 14, mean 5,330,000 cp/mL) according to Livant et al. [11] and 500,000–32,000,000 cp/mL (n = 16, mean 5,700,000) according to Mourez et al. [3]. Sirivichayakul et al. reported three Ag-positive cases with copy numbers of 100,000 cp/mL, and 23 other Ag-positive and 6 Ag- and Ab-positive cases with copy numbers >1,000,000 cp/mL [12]. HIV-1 QL-NAT detection sensitivity was sufficient to detect these Ag-positive cases.

When analyzing commercial HIV-1 seroconversion/performance panels, three samples that were HIV-1-positive using Determine HIV Early Detect reacted with the HIV-2 gp140 line in the HIV Ab confirmatory test using Geenius HIV-1/2 confirmatory assay, resulting in HIV-2 indeterminate or HIV indeterminate status, despite being on the HIV-1 seroconversion/performance panel. Livant et al. reported that two of 16 pre-seroconverted specimens that were HIV-1 RNA-positive were also HIV-2 gp140-reactive [11]. In our previous study, four of 89 (4.5 %) HIV-positive samples cross-reacted with the HIV-2 gp140 line, although all were identified as HIV-1-positive when analyzed using Geenius Reader [13]. Thus, HIV-2 gp140-reactive cases could be related to the cross-reactivity of anti-HIV-1 Abs or nonspecific reactions. The HIV-1/2 differentiation test is important for determining treatment strategies for infected patients [14]. Even if anti-HIV-2 Ab cross-reactivity is suspected based on the HIV Ab confirmatory test, the HIV-1 QL NAT may be useful for HIV-1/2 differentiation.

As the conditions for in-house testing vary depending on the reagents and equipment used, performing precision management in each testing laboratory is necessary when using the method established in this study for HIV testing. A validation test using positive and negative controls should be performed each time, while confirming the uniformity between the threshold cycle (Ct) value of the positive control as measured by the intercalator method and concentration of the band as observed by agarose gel electrophoresis. In addition, the lower LODs of Ag detection using the ICA method and HIV-1 QL-NAT based on the RNA copy number differed by 2 log or more. Although not experienced in our study, the possibility of false positives should be considered in confirmatory test of HIV-1 Ag-positive specimens using ICA when higher values are observed in the intercalator method or when a band thinner than the positive control is observed by agarose electrophoresis in the HIV-1 QL-NAT. The use of this method is only advisable as an adjunct confirmatory test for HIV-1 Ag-positive and HIV-1 Ab confirmatory test-indeterminate or -negative cases.

From the data of the AIDS Surveillance Committee in Japan, the number of HIV-infected patients, HIV consultations, and diagnostic tests has decreased [1]. Ejima et al. report that the number of HIV-infected patients who were not diagnosed with AIDS did not decrease as much as the number of HIV tests during the COVID-19 pandemic [15]. Populations with high-risk behavior might be concerned about HIV infection even during this period. On the other hand, populations with low-risk behavior might be discouraged from HIV testing because of reduced opportunities for potential HIV infection secondary to movement restrictions and/or HIV tests discontinuation. In addition, in urban areas with high HIV prevalence, AIDS and HIV awareness activities are conducted and there are many HIV testing facilities and opportunities, whereas in rural areas with low HIV prevalence, limited testing personnel and infrastructure were redirected to COVID-19 testing. It might also reflect the loss of testing opportunities.

The proportion of newly-reported AIDS patients in 2021 was higher in most rural prefectures than in urban prefectures [1], suggesting that more HIV-infected patients might be missed in rural area. This trend has been observed prior to the COVID-19 pandemic. It is important to expand HIV testing opportunities and establish a testing system in rural areas so that treatment can be initiated as early as possible after infection. On the other hand, it is not easy to establish testing facilities with expensive equipment and IVDs in rural areas with low HIV prevalence. Public health centers and prefectural diagnostic laboratories in Japan provide NAT for various infectious diseases for which no approved IVDs exist. The qualitative HIV-1 NAT discussed in this study can be performed using the same technology, and should be useful as an adjunct confirmatory test for HIV-1 Ag-positive cases by HIV ICA or for HIV screening test-positive and HIV Ab confirmatory test-negative or -indeterminate cases in these laboratories.

## 5 Conclusion

We established a simple and inexpensive HIV-1 QL-NAT for use as an adjunct HIV-1 confirmatory test for HIV-1 Ag-positive specimens using Determine HIV Early Detect. These methods are useful for confirming HIV-1 Ag-positive cases and excluding HIV-1 Ag-false-positive cases in low-HIV-prevalence areas and small- and medium-sized diagnostic laboratories.

## Data availability statement

The authors confirm that the data supporting the findings of this study are available within the article and its supplementary materials.

## Ethics approval

Ethics approval was obtained from the Ethical Committee of the National Institute of Infectious Diseases (No. 1082).

## Funding

This work was supported by the 10.13039/501100003478Ministry of Health, Labour and Welfare (10.13039/501100003478MHLW) of Japan (Health and Labour Sciences Research Grants [19HA1001 and 22HA2001]).

## CRediT authorship contribution statement

**Shigeru Kusagawa:** Writing – review & editing, Writing – original draft, Visualization, Validation, Software, Resources, Project administration, Methodology, Investigation, Formal analysis, Data curation, Conceptualization. **Ai Kawana-Tachikawa:** Writing – review & editing, Supervision, Resources, Project administration, Funding acquisition, Conceptualization. **Saori Matsuoka:** Writing – review & editing, Supervision, Resources, Project administration, Methodology, Investigation, Funding acquisition, Formal analysis, Conceptualization.

## Declaration of competing interest

The authors declare that they have no known competing financial interests or personal relationships that could have appeared to influence the work reported in this paper.

## Figures and Tables

**Figure:**
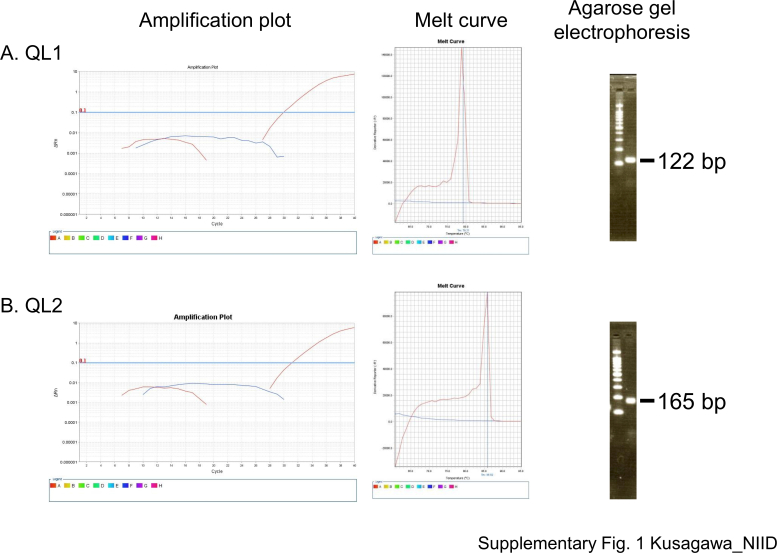


**Figure:**
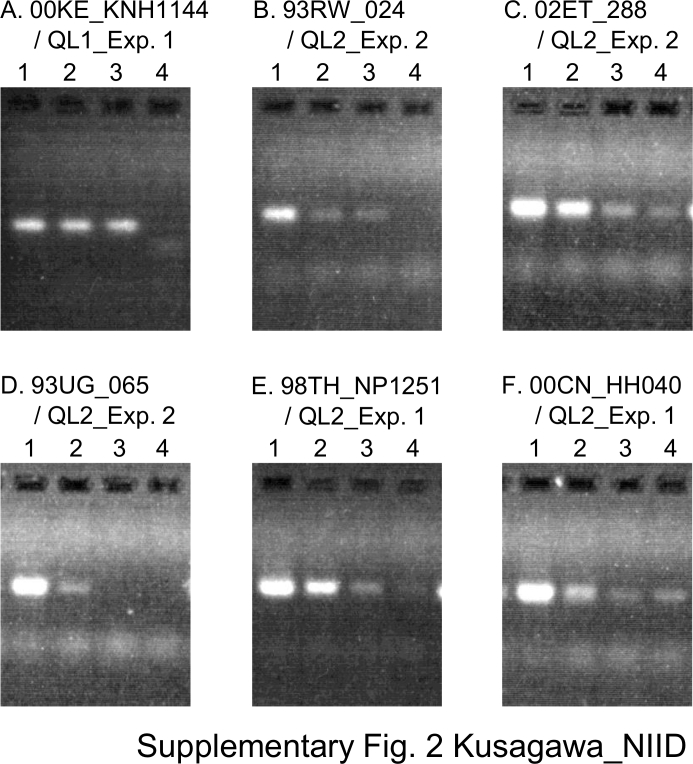


**Figure:**
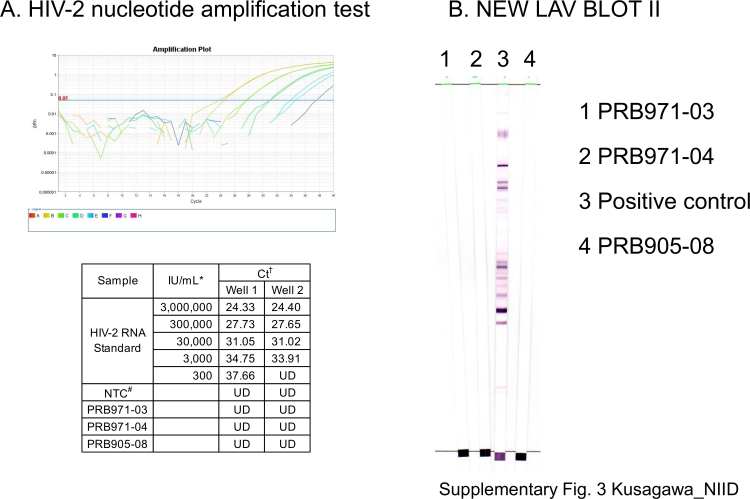


## Abbreviations

HIV: human immunodeficiency virus
AIDS: acquired immunodeficiency syndrome
COVID-19: coronavirus disease 2019
ICA: immunochromatography assay
Ab: antibody
Ag: antigen
IVDs: *in vitro* diagnostics
NAT: nucleotide amplification test
HIV-1 QL-NAT: in-house qualitative HIV-1 NAT
BM53: Base Matrix 53
cp/mL: copies/mL
LOD: limit of detection
CRF: circulating recombinant form
HIV-1 types: HIV-1 subtypes, circulating recombinant forms, and groups
Tm: melting temperature
Ct: threshold cycle
