# Supplementary material for: Establishment of qualitative human immunodeficiency virus type 1 nucleic acid amplification test as an adjunct confirmatory test in low-prevalence areas and small- and medium-sized diagnostic laboratories
Source: Heliyon. 2024 Jan 13;10(2):e24451. doi: 10.1016/j.heliyon.2024.e24451 (PMC10831601; doi:10.1016/j.heliyon.2024.e24451)
Supplement: Supplementary file 1 [file mmc1.docx]

Supplementary Table 1. QL1 and QL2 PCR primers and reaction cycle

| QL1 | | |
| --- | --- | --- |
| Primers | Forward (580A) | 5ʹ-GAT GGG TGC GAG AGC GTC-3ʹ (789-806 HXB2) |
|  | Reverse (612B) | 5ʹ-GCT CCC TGC TTG CCC ATA CTA-3ʹ (910-890 HXB2) |
|  | Fragment size | 122 bp (HXB2) |
| Cycle | RT | 50 °C 15 min > 95 °C 10 min |
|  | PCR | (95 °C 10 s > 60 °C 15 s > 72 °C 30 s) × 40 cycles |
|  | Dissociation | 95 °C > 60 °C > 95 °C (1 °C/min) |
| QL2 | | |
| Primers | Forward (Gag183UF) | 5ʹ-CTA GCA GTG GCG CCC GAA CAG-3ʹ (629-649 HXB2) |
|  | Reverse (Gag187LR) | 5ʹ-CCA TCT CTC TCC TTC TAG CCT CCG CTA GTC A-3ʹ (793-763 HXB2) |
|  | Fragment size | 165 bp (HXB2) |
| Cycle | RT | 50 °C 15 min > 95 °C 10 min |
|  | PCR | (95 °C 10 s > 72 °C 45 s) × 40 cycles |
|  | Dissociation | 95 °C > 60 °C > 95 °C (1 °C/min) |

Supplementary Table 2. QL1 and QL2 detection sensitivity evaluation using ACCURUN315

| Sample | QL1 | | | | | | QL2 | | | | | | COBAS^‡^ |
| --- | --- | --- | --- | --- | --- | --- | --- | --- | --- | --- | --- | --- | --- |
|  | Exp. 1 | | | Exp. 2 | | | Exp. 1 | | | Exp. 2 | | |  |
|  | Ct^†^ | Tm^\^ | EP^#^ | Ct | Tm | EP | Ct | Tm | EP | Ct | Tm | EP | cp/mL |
| 315_500 | 25.56 | 78.83 | + | 25.36 | 78.84 | + | 27.46 | 88.30 | + | 27.34 | 86.80 | + | 170,000 |
| 315_400 | 28.73 | 79.88 | + | 29.16 | 78.84 | + | 30.83 | 88.30 | + | 30.95 | 88.31 | + | 20,000 |
| 315_300 | 30.65 | 79.88 | + | 31.50 | 79.88 | + | 33.83 | 88.30 | + | 34.36 | 88.31 | + | 3,500 |
| 315_200 | 32.46 | 79.88 | + | 33.16 | 79.88 | + | 35.09 | 88.30 | + | 35.33 | 88.31 | + | 820 |
| 315_100 | UD^¶^ | 60.76 | - | UD | 60.76 | - | UD | 61.25 | - | UD | 61.26 | - | 57 |
| BM53^§^ | UD | 60.76 | - | UD | 60.76 | - | UD | 61.25 | - | UD | 62.33 | - | NT^$^ |

Ct^†^, Threshold cycle; Tm^\^, Melting temperature; EP^#^, agarose gel electrophoresis; BM53^§^, BaseMatrix53; UD^¶^, undetermined; COBAS^‡^, cobas HIV-1 quantitative; NT^$^, not tested
